# Risk of Dementia in Immunocompromised and Non-immunocompromised Persons with COVID-19 Infection

**DOI:** 10.1093/geroni/igaf122.3649

**Published:** 2025-12-31

**Authors:** Winnie Xu, Yonah Ziemba, James Crawford, Michael Diefenbach, Shahidul Islam, Cristina Sison, Christian Nouryan, Edith Burns

**Affiliations:** Hofstra University, Hempstead, New York, United States; Northwell, Manhasset, New York, United States; Northwell, Manhasset, New York, United States; Northwell, Manhasset, New York, United States; Northwell Health, New Hyde Park, New York, United States; Northwell, Manhasset, New York, United States; Northwell Health, Great Neck, New York, United States; Zucker School of Medicine Hofstra-Northwell, Manhasset, New York, United States

## Abstract

COVID-19 infection is associated with an increased risk of developing dementia, particularly among older adults. We examined the impact of immune status on this risk using the COVID-19 Real World Data Infrastructure (DOI: 10.1093/0fid/ofaf021), a publicly available resource of 5.2 million unique deidentified patient records, linked with the NCI SEER registry. Two cohorts of patients were defined for the study period (12/1/2018-12/31/2024): COVID-19 Positive Persons (CPP) with positive SARS-CoV-2 NAAT and/or COVID-19 ICD-10 code. COVID-19 Negative Persons (CNP) had COVID-related information (including SARS-CoV-2 diagnostic or serologic testing and/or documented vaccination), but no positive NAAT or COVID-19 ICD-10 code. Patients < 65 years, with history of dementia or psychotropic medication use on or before index date (1/1/2020) were excluded. There were 225,852 CPP and 499,295 CNP eligible patients. Incidence of newly-diagnosed dementia among the CPP cohort was 25.6%, vs. 17.3% in CNP (Relative Risk [RR] 1.48; 95% CI: 1.46-1.49). Stratification by immunocompromised status (473,559 immunocompromised; 251,588 non-immunocompromised) showed no significant difference between RR for dementia associated with COVID-19 (RR = 1.48, 95% CI:1.46-1.50 and RR = 1.47, 95% CI: 1.45-1.50 respectively). These unadjusted analyses align with prior findings that COVID-19 infection in patients aged ≥65 years is associated with a greater risk of subsequent dementia diagnosis but suggest immunocompromised individuals have the same increased risk as the general population. Further analysis will examine timing of diagnoses, and relevance of covariates such as vaccination status and anti-viral medication use on dementia risk in CPP patients.

